# QTL Map of Early- and Late-Stage Perennial Regrowth in *Zea diploperennis*

**DOI:** 10.3389/fpls.2021.707839

**Published:** 2021-08-24

**Authors:** Kyle W. Swentowsky, Harrison S. Bell, David M. Wills, R. Kelly Dawe

**Affiliations:** ^1^Department of Plant Biology, University of Georgia, Athens, GA, United States; ^2^Department of Genetics, University of Georgia, Athens, GA, United States

**Keywords:** perennial, maize, *Zea diploperennis*, QTL, tillers

## Abstract

Numerous climate change threats will necessitate a shift toward more sustainable agricultural practices during the 21st century. Conversion of annual crops to perennials that are capable of regrowing over multiple yearly growth cycles could help to facilitate this transition. Perennials can capture greater amounts of carbon and access more water and soil nutrients compared to annuals. In principle it should be possible to identify genes that confer perenniality from wild relatives and transfer them into existing breeding lines to create novel perennial crops. Two major loci controlling perennial regrowth in the maize relative *Zea diploperennis* were previously mapped to chromosome 2 (*reg1)* and chromosome 7 (*reg2*). Here we extend this work by mapping perennial regrowth in segregating populations involving *Z. diploperennis* and the maize inbreds P39 and Hp301 using QTL-seq and traditional QTL mapping approaches. The results confirmed the existence of a major perennial regrowth QTL on chromosome 2 (*reg1*). Although we did not observe the *reg2* QTL in these populations, we discovered a third QTL on chromosome 8 which we named *regrowth3 (reg3)*. The *reg3* locus exerts its strongest effect late in the regrowth cycle. Neither *reg1* nor *reg3* overlapped with tiller number QTL scored in the same population, suggesting specific roles in the perennial phenotype. Our data, along with prior work, indicate that perennial regrowth in maize is conferred by relatively few major QTL.

## Introduction

Plant growth habits fall into one of two categories. Annual species undergo their complete life cycle which involves germination, vegetative growth, reproduction, and senescence, in one year. Perennials exhibit these same growth stages but do not fully senesce and are capable of new vegetative growth following senescence. Some perennial species are seasonally dormant while others stay green year round. Evolutionary transitions between annual and perennial growth modes have occurred numerous times during land plant evolution (Friedman and Rubin, 2015; Lindberg et al., 2020). Perenniality is usually the ancestral trait and the annual growth mode is derived (Friedman and Rubin, 2015). For instance, a transition from perennial to annual growth may have allowed plants to survive seasonal stresses such as drought (Sherrard and Maherali, 2006; Friedman and Rubin, 2015). Taxa that allocate relatively more resources to above-ground organs have been more likely to evolve annuality (Lindberg et al., 2020).

Although our major cereal crops today are annuals, it has been hypothesized that growing perennial grains could result in sustainable agriculture benefits (Zhang et al., 2011; Fernando et al., 2018). Compared to annuals, perennials grow deeper, more established root systems that allow them to tap into water or nutrients further below the soil surface. Perennials can also emerge earlier in the growing season which allows them to capture more light (Dohleman and Long, 2009). The increased photosynthesis could theoretically lead to higher productivity while mitigating effects of climate change through more effective capture and sequestration of carbon dioxide.

New perennial cereal crops must be developed to recognize the sustainable benefits of perennial agriculture (Crews and Cattani, 2018). One way to achieve this goal would be to select for domestication traits in populations of an existing perennial species. A new cereal trademarked as Kernza^®^ has been developed from the intermediate wheatgrass species *Thinopyrum intermedium* using this approach. Some of the hypothetical benefits of perennial agriculture have been achieved with Kernz^®^ (de Oliveira et al., 2018, 2020), but its yield is currently much lower than that of wheat (Culman et al., 2013). Perennial grains generally have lower natural yield than congeneric annuals (Vico et al., 2016), and will likely require intensive long-term selection to improve yields.

An alternative approach is to breed perenniality into existing annual species (Murray and Jessup, 2014). It may be possible to leverage the already high yields of modern cultivars while bringing in alleles that provide some degree of perenniality. Several groups have initiated these efforts by focusing on the formation of rhizomes as a proxy for perennial regrowth. Rhizomes are below-ground organs derived from stems that can store resources used for regrowth. Trait mapping studies in *Oryza* (Hu et al., 2003; Fan et al., 2020), *Sorghum* (Paterson et al., 1995; Kong et al., 2015), and *Zea* (Westerbergh and Doebley, 2004) revealed QTL associated with the presence of rhizomes in F_2_ progeny. These are important first steps but it remains unclear whether lines bred for rhizome formation will demonstrate perennial regrowth. A recent study took a different approach of mapping genes that promote regrowth itself by crossing domesticated maize to its close perennial ancestor of maize (*Zea mays* L.) called *Zea diploperennis* (Iltis et al., 1979; Ma et al., 2019). Regrowth is generally scored as the presence of new branches at the base of the plant after the plant has flowered and senesced. The authors found two *Zea* regrowth QTL termed *regrowth1* (*reg1*) and *regrowth2* (*reg2*) and noted that these are distinct from previously found rhizome QTL in *Z. diploperennis*. The authors noted that rhizomes are almost never found in their regrown plants and that regrowth can occur from dormant tiller buds.

Here we report the results from mapping regrowth in two F_2_ populations between *Z. diploperennis* and maize inbred lines with high tillering. We found two genomic regions associated with regrowth using a modified bulk segregant analysis approach called QTL-seq (Michelmore et al., 1991; Takagi et al., 2013) in combination with targeted PCR-based genotyping. One QTL maps to the same location as *reg1* (Ma et al., 2019), while a second, previously unreported QTL maps to chromosome 8 and shows a strong effect in the later stages of regrowth.

## Materials and Methods

### Plant Materials, Growth Conditions, and Phenotyping

**FIGURE 1:**
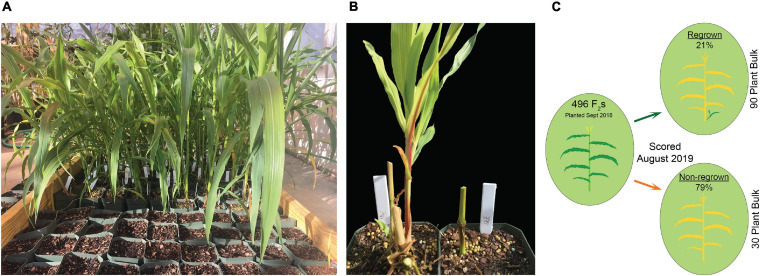
Growth conditions for scoring regrowth in F_2_ populations. **(A)** Seedlings were grown in square four-inch pots over a moist coconut mat. **(B)** Following flowering and senescence, regrown (left) and non-regrown (right) plants were scored. **(C)** Schematic for QTL-seq experiment. 496 P39/“Gigi” F_2_s were planted in September 2018 and scored in August 2019 for regrowth (21%; top) and non-regrowth (79%; bottom). QTL-seq was carried out on a 90-plant regrown bulk and a 30-plant non-regrown bulk.

*Zea diploperennis* “Gigi” is the clone of a plant grown from seed obtained from Germplasm Resources Information Network (GRIN), Ames, IA, United States (PI 462368). F_2_ populations were generated by crossing pollen from “Gigi” to ears of the maize sweetcorn line P39 (PI 587133) and popcorn line Hp301 (PI 587131). Approximately five F_1_s from each family were sown in isolation and allowed to intermate freely to generate F_2_ seeds. The F_2_ progeny were analyzed in three separate experiments. Two experiments were carried out entirely in a greenhouse environment by growing progeny in densely placed four-inch pots placed over moist coconut mats (Figure 1). One greenhouse experiment was started in the fall of 2018 and involved only the P39/“Gigi” population, and the second greenhouse experiment was started in the fall of 2019 and included both the P39/“Gigi” and Hp301/“Gigi” populations. At the time of the second greenhouse experiment 2019 we also planted a population of P39/“Gigi” F_2_ individuals in a UGA field plot where we measured regrowth and tillering before a hard freeze ended the experiment.

For greenhouse experiments, seeds were sown in square four-inch pots using a pine bark soil mixture supplemented with Osmocote slow-release fertilizer. Approximately one-third of seeds germinated. Pots with germinated seedlings were placed close together on top of a coconut mat. They were watered daily and roots were allowed to grow into the coconut mat. The first experiment (for QTL-seq) used 496 P39/“Gigi” F_2_s and began in September 2018. A second experiment (for QTL mapping using PCR primers) involved 196 P39/“Gigi” F_2_s and 419 Hp301/“Gigi” F_2_s and began in September 2019. In both cases, plants flowered in November and December then began to senesce. Fully senesced (brown) stems were cut back to the soil line. Positive regrowth was scored by the presence of green leaves on young tillers. Regrowth was scored twice in the QTL-seq experiment during April and August 2019. In the subsequent QTL mapping experiment, regrowth was scored on four dates in 2020: January 23, February 19, March 24, and May 6.

Field-grown plants were initially sown in pine bark soil in September 2019 and then 227 P39/“Gigi” F_2_s were transplanted into a field site in Athens, GA approximately 2 weeks after sowing. Plants were then treated with Osmocote slow-release fertilizer and kept watered. Total tiller number and regrowth (defined in this case as a binary trait by the presence of tillers initiated post-senescence) were scored on December 1, 2019 before a hard freeze on December 2 killed all plants.

### DNA Extraction and Illumina Library Preparation/Sequencing

Regrowth (RG) and non-regrowth (NRG) bulks were prepared by pooling leaves of equal sizes from 90 and 30 plants per bulk, respectively. Tiller number bulks were created by pooling leaves of equal sizes from 26 plants per bulk using plants with 0–2 tillers (low tiller) and 9–15 tillers (high tiller). Bulk DNA was extracted from each of the four bulks using the Genomic DNA Mini Kit (Plant) (IBI, cat no. IB47231). Genomic DNA from the *Z. diploperennis* parent “Gigi” was extracted using a single leaf also using the Genomic DNA Mini Kit (Plant) (IBI, cat no. IB47231). Libraries for Illumina sequencing of these four bulks and “Gigi” genomic DNA were generated using the KAPA HyperPrep Kit for NGS (Roche, cat no. KK8502). The number of Illumina reads per sample are shown in Supplementary Table 1. The NRG bulk library was sequenced twice on two separate flow cells and the resulting reads were combined for analysis. All reads were deposited in the NCBI Sequence Read Archive under BioProject PRJNA700589.

### SNP Calling and QTL-Seq Analysis

QTL-seq analysis is a bulk segregant analysis approach employing SNPs identified via next-generation sequencing methods. Two separate DNA bulks are made from 20 to 50 or so offspring with the opposite extremes for the phenotype of interest. QTL are determined as genomic regions in which parental SNP frequencies deviate significantly from the expected Mendelian ratios in the DNA bulks. The GATK Best Practices Pipeline for Germline short variant discovery was applied to call SNPs and determine SNP-index from Illumina data (DePristo et al., 2011; Poplin et al., 2017). Briefly, reads were mapped to the P39 reference (Hufford et al., 2021) using BWA MEM v0.7.15 (Li, 2013), sorted using SAMtools sort v1.3.1 (Li et al., 2009), and duplicate reads were marked using Picard^1^. HaplotypeCaller (GATK/4.0.11.0) using default settings followed by GenotypeGVCFs were used to call SNPs from “Gigi,” RG, and NRG samples and 34,235,573 raw SNPs were identified. SelectVariants was used to filter SNPs based on all of the following criteria: homozygous in “Gigi” with depth (DP) of at least three reads; Genotype Quality (GQ) of at least 99 in RG and NRG. This process retained 2,180,252 SNPs. The filtered VCF file was exported using the VariantsToTable command for analysis using RStudio v1.2.1335. SNPs for high tillered (HT) and low tillered (LT) bulks were called independently with the same workflow and only homozygous “Gigi” SNPs were retained for SNP-index analysis (Supplementary Figure 1 and Supplementary Table 2).

Allele frequency of each SNP in each bulk was calculated using RStudio by dividing the number of reads supporting a SNP by the number of reads covering the position of the SNP (i.e., AD/DP). The genome was divided into 1 Mb bins and the mean SNP-index was calculated for each bin. This bin size was chosen since it provided sufficient QTL-seq approximation while allowing for the data to be clearly displayed. The SNP-index and ΔSNP-index (RG – NRG or HT – LT) were plotted according to their respective genomic locations using ggplot2 (Wickham, 2016).

### PCR Genotyping

Genomic DNA was extracted from leaves using a modified version of the method described by Saghai-Maroof et al. (1984). Leaves were flash frozen in liquid nitrogen and ground with a pestle and mortar. Ground tissue was suspended in CTAB solution (100 mM Tris, 700 mM NaCl, 50 mM EDTA, 1% CTAB, 140 mM B-mercaptoethanol) and heated at 65°C for 1 h. Aqueous components were extracted by mixing this solution with chloroform/isoamyl alcohol and centrifugation. Nucleic acids were isolated from the aqueous layer by isopropanol precipitation and the pellet was cleaned using 70% ethanol. The pellet was suspended in 100 μl TE.

Genomic DNA was aliquoted into 96-well plates for high-throughput PCR using GoTaq Green Master Mix (Promega, cat no. M7123). A final primer concentration of 1 μM was used to generate amplicons across polymorphic sites. We used a draft “Gigi” genome assembly (generously provided by Matthew Hufford, Iowa State University) to design primers that capture indels or SNPs on chromosomes 2, 7, and 8 that differentiate “Gigi” from P39 or Hp301. A total of 22 and 20 markers were designed to capture *Z. diploperennis* polymorphisms against P39 and Hp301, respectively (Supplementary File 1). The majority of markers captured indels, however, two were CAPS markers that required digestion with the restriction enzyme *Eco*RV-HF (NEB, cat no. R3195T). A full list of primer sequences and genomic positions are provided in Supplementary File 1.

For the 2018 greenhouse trial, 96 regrown P39/“Gigi” F_2_s of the 496 total were genotyped for three markers. Data was obtained for DCP23/DCP24 on chromosome 2 (92 plants), DPP7/DPP8 on chromosome 7 (94 plants), and DCP84/DCP85 on chromosome 8 (96 plants). For the 2019 greenhouse trial, we genotyped all 196 of the P39/“Gigi” F_2_ plants, however, we only genotyped 40 regrown and 152 non-regrown individuals from the population of 419 Hp301/“Gigi” F_2_ plants. For the 2019 field trial, we genotyped 35 regrown and 71 non-regrown plants from the population of 227 P39/“Gigi” F_2_ individuals.

### QTL Analysis

QTL analysis was carried out using the R package R/qtl (Broman et al., 2003). Data for the P39/“Gigi” F_2_ population (Supplementary File 2) or Hp301/“Gigi” F_2_ population (Supplementary File 3) were imported using the read.cross() function with the option map.function = “kosambi.” Individuals with data for fewer than 11 (P39/“Gigi”) or 8 (Hp301/“Gigi”) total markers were removed. Segregation distortion was analyzed using the geno.table() function, however, no markers showing significant segregation distortion were found. The markers were placed in the order they occur in the P39v1 physical map (Hufford et al., 2021) and genetic map distances were estimated with the est.rf() function. The genetic distances were consistent with the ordering on the physical map. Individuals showing excessively large numbers of crossovers (P39/“Gigi” >7 and Hp301/“Gigi” >6) were removed from the analysis. With these individuals removed, the genetic map was calculated again using the est.rf() function. Linkage between genotypic data and phenotypes (regrowth scored during January, February, March, and May) was calculated using the scanone() function with options method = “em” and model = “binary.” QTL analysis was performed using the scanone function in R/qtl using the binary model and maximum likelihood estimation (Xu and Atchley, 1996; Broman et al., 2003). LOD score data for each marker were organized into a new data frame that incorporated each marker’s physical position and plotted using ggplot2 (Wickham, 2016).

## Results

To screen for genetic loci associated with perennial regrowth in *Zea*, we generated two F_2_ mapping populations. Pollen from a *Z. diploperennis* clone named “Gigi” was crossed onto ears from two maize inbred lines: P39 (a sweetcorn) and Hp301 (a popcorn). We chose P39 and Hp301 because they generate many tillers (Kebrom and Brutnell, 2015; Zhang et al., 2019), which we hypothesized might increase the penetrance of perennial phenotypes. Around five F_1_s resulting from each cross were allowed to intermate in isolation and produce F_2_ seed.

### Greenhouse Studies of Regrowth

**FIGURE 2:**
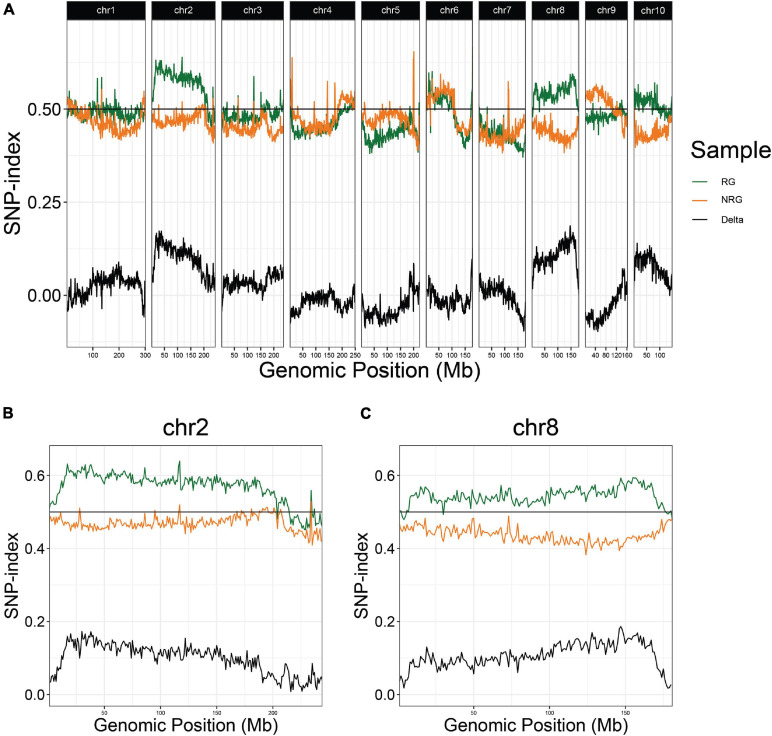
QTL-seq for regrowth reveals two major loci in a P39/“Gigi” F_2_ population. **(A)** Average SNP-index per 1 Mb window is plotted across all ten chromosomes. Chromosomes 2 **(B)** and 8 **(C)** are shown in greater detail. For each plot, lines display SNP-index of regrown (RG) bulk in green, non-regrown (NRG) bulk in orange, and the difference between the two bulks (RG – NRG) in black.

In the first experiment, planted in September of 2018, 496 plants flowered in November–December and the upper parts of the plants senesced to turn brown. Following this first cycle, 203 (41%) the plants grew fresh stems that eventually flowered in early 2019. Another round of senescence occurred in spring of 2019, followed by regrowth in 104 (21%) of the original 496 plants (Table 1 and Supplementary File 4). After second flowering, we performed a QTL-seq experiment comparing a bulk of 90 regrown and 30 non-regrown bulks to determine which genomic regions were associated with regrowth. Two loci displayed higher SNP-indices in the RG compared to the NRG bulk (Figure 2). The first is on the short arm of chromosome 2, and corresponds to the dominant QTL previously identified as *regrowth1* (*reg1*) (Ma et al., 2019). The second is located on the long arm of chromosome 8 near position 150 Mb on the P39 physical map, and will be referred to as *regrowth3* (*reg3*). The *regrowth2* locus on the short arm of chromosome 7 (Ma et al., 2019) did not show significant differences in SNP-index between the two sequenced bulks.

**TABLE 1:** Number of regrown (RG) and non-regrown (NRG) plants in P39/“Gigi” F_2_ greenhouse-grown plants planted in 2018 and scored after one and two cycles of regrowth in 2019.

	First regrowth, April 2019	Second regrowth, August 2019
RG	203	104
NRG	293	392
Percent RG	40.9%	21.0%

*All plants that did not regrow during the first cycle also did not regrow in the second cycle.*

The bulk sequencing results were confirmed on individual plants by designing codominant PCR markers spanning polymorphisms near each of the SNP-index peaks. The markers were scored in a sample of 96 regrown plants from the same 2018 population. The *Z. diploperennis* markers for *reg1* and *reg3* were represented at frequencies significantly higher than Mendelian expectations. An excess of heterozygotes were observed at both loci indicating dominant inheritance patterns (Table 2 and Supplementary File 5). There was also a low number of RG individuals that were homozygous for the P39 allele suggesting that *reg1* and *reg3* are not fully penetrant or that other loci can compensate for their function. Only 1 of 92 RG individuals positively genotyped at these loci were homozygous for the P39 allele of both *reg1* and *reg3*. The marker near *reg2* on chromosome 7 segregated at a 1:2:1 Mendelian ratio, consistent with the SNP-index data that failed to show evidence that *reg2* contributes to regrowth in the P39/“Gigi” population (Table 2). These results support the existence of two *Z. diploperennis* alleles that co-segregate with RG plants in our population: the previously described *reg1* and the previously unknown *reg3*.

**TABLE 2:** Genotype distribution of markers in greenhouse-regrown P39/“Gigi” F_2_ plants.

Genotype	chr2 (44.6 Mb)	chr7 (4.5 Mb)	chr8 (139.5 Mb)
Gigi/Gigi	30	18	43
Gigi/P39	55	44	41
P39/P39	7	32	12
χ^2^	15.022	4.254	22.063
*p*	**0.0005**	0.1192	**<0.0001**

*Numbers of plants with specified genotype (rows) at each genomic marker (columns) are displayed. Bottom rows depict chi-square and p-values to analyze significant deviation from Mendelian (1:2:1) expectations. p-values below 0.05 are bold.*

**FIGURE 3:**
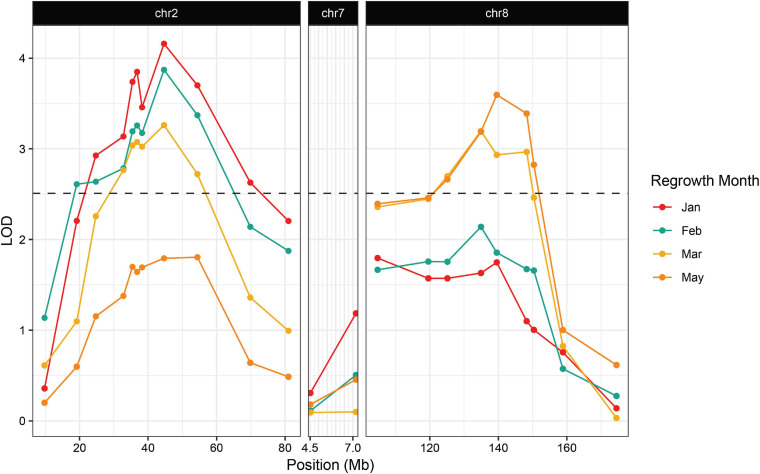
Association between regrowth and markers on chromosomes 2, 7, and 8 in the 2019 P39/“Gigi” F_2_ population. QTL were considered significant if the LOD score exceeded the 95% threshold determined by 1000 random permutations of the data for each trait. The threshold was calculated independently for each time point. February had the highest threshold of LOD = 2.51 and was used here as the significance cutoff (dashed line).

We planted a second wave of individuals in September 2019, but instead of a single late observation, plants were scored for green tissue at four time points: once in January, February, March, and May. This time course revealed that early regrowth does not necessarily translate to continued regrowth. A number of plants that showed evidence of regrowth early in the season ultimately senesced and died. In January we scored 42.3% of the plants as regrown, whereas in May, only 25% of the plants were still flourishing (Table 3). PCR genotyping revealed that markers linked to *reg2* on chromosome 7 showed no significant association with regrowth at any time point (Figure 3 and Supplementary File 2). However, we observed significant associations between regrowth and markers on chromosomes 2 (*reg1*) and 8 (*reg3*). The highest linkage was between regrowth scored in January and a marker on chromosome 2 (at 44.6 Mb) with a LOD of 4.10. Association between regrowth and the chromosome 2 marker dropped during subsequent sampling points, and there was no significant linkage detected at the latest time point in May. In marked contrast, a marker on chromosome 8 at 139.6 Mb became more significant at later sampling points, such that significant LOD scores were only observed in March and May (with a LOD of 3.37). These data suggest that *reg1* is important for the initiation of regrowth while *reg3* has a more significant impact later in the growth cycle.

**TABLE 3:** Numbers of regrown (RG) and non-regrown (NRG) plants in F_2_ populations scored during different time points following flowering and senescence in 2019.

	P39/“Gigi” F_2_s			Hp301/“Gigi” F_2_s		
Date scored	RG	NRG	Percent RG	RG	NRG	Percent RG
January	83	113	42.3%	35	384	8.4%
February	66	130	33.7%	43	376	10.3%
March	61	135	31.1%	33	386	7.9%
May	50	146	25.5%	27	392	6.4%

**FIGURE 4:**
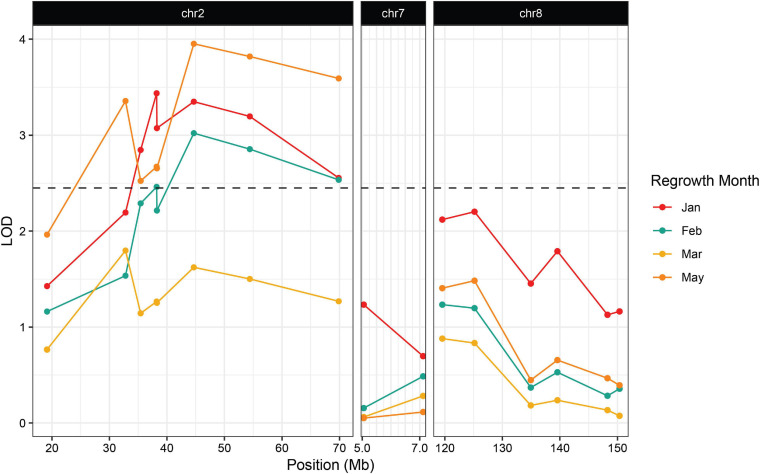
Association between regrowth and markers on chromosomes 2, 7, and 8 in the 2019 Hp301/“Gigi” F_2_ population. QTL were considered significant if the LOD score exceeded the 95% threshold determined by 1000 random permutations of the data for each trait. The threshold was calculated independently for each time point. May had the highest threshold of LOD = 2.45 and was used here as the significance cutoff (dashed line).

The second wave planting also included plants from the Hp301/“Gigi” F_2_ mapping population. The observed regrowth rate was considerably lower for this group, ranging from 6.4 to 10.3% (Table 3). A total of 192 Hp301/“Gigi” F_2_ individuals were genotyped, including 40 plants that showed regrowth during at least one time point. Significant association was not observed between regrowth and any markers on chromosomes 7 or 8, suggesting that neither *reg2* or *reg3* segregate in the Hp301/“Gigi” F_2_ population (Figure 4 and Supplementary File 3). In contrast, several chromosome 2 markers were significantly associated with regrowth, with the highest LOD score of 3.95 between the marker at 44.6 Mb and regrowth scored in May. We observed the highest association in the Hp301/“Gigi” population when regrowth was scored in May, second highest in January, third highest in February, and non-significant association in March (Table 3). To test for the possibility of linkage disequilibrium between *reg1* and *reg3*, we compared the genotypes at the two QTL in both P39/“Gigi” and Hp301/“Gigi” F_2_ populations using a Chi-squared test for linkage. We did not observe LD between the QTL that could potentially lead to spurious trait associations (χ^2^ *p* > 0.05; df = 5).

### Field Studies of Regrowth and Tillering

An outdoor field planting of the P39/“Gigi” population was carried out in the fall of 2019, both to confirm the mapping data and to test whether the plants could survive a winter freeze and dormant season. P39/“Gigi” F_2_ individuals were planted in September, and flowered and began to senesce by mid to late-November before a hard freeze in early December. At the time of the freeze, 38/228 (16.7%) of the plants had regrown. 35 regrown and 71 non-regrown plants were genotyped for a single marker on chromosomes 2, 7, and 8 (Table 4 and Supplementary File 6). The only significant deviation from Mendelian expectations was observed on chromosome 2 (*reg1*), where regrown plants were significantly enriched for the “Gigi” allele. This is consistent with our greenhouse studies, since *reg3* was only observed later in the growth cycle. The field site was monitored for the next 5 months, but no plants survived to regrow the next spring.

**TABLE 4:** Genotype distribution of markers in field-grown P39/“Gigi” F_2_ plants either showing regrowth (RG) or no regrowth (NRG).

	chr2 (44.6 Mb)		chr7 (4.5 Mb)		chr8 (139.5 Mb)	
Genotype	RG	NRG	RG	NRG	RG	NRG
“Gigi”/“Gigi”	14	15	6	22	7	20
“Gigi”/P39	18	43	19	35	15	29
P39/P39	3	13	10	14	13	22
χ^2^	6.837	3.717	1.346	1.778	2.458	2.140
*p =*	**0.0328**	0.1559	0.5101	0.4111	0.2927	0.3431

*Bottom rows depict chi-square and p-values to analyze significant deviation from Mendelian (1:2:1) expectations. p-values below 0.05 are bolded.*

**FIGURE 5:**
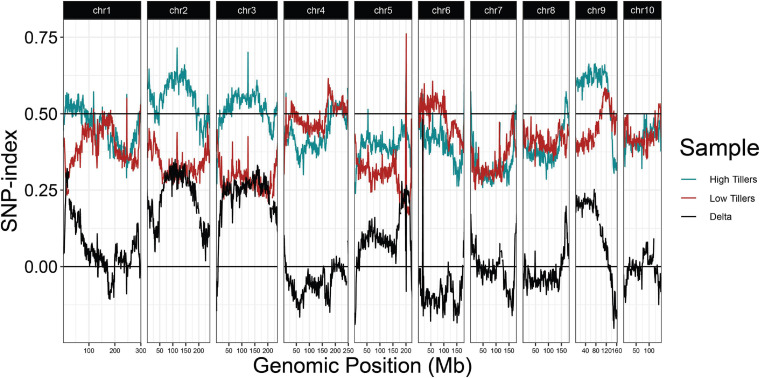
QTL-seq for tiller number supports numerous tillering QTL in the 2019 P39/“Gigi” F_2_ field population. Average SNP-index per 1 Mb window is plotted across all ten chromosomes. Lines display SNP-index in high (blue) and low (red) tiller bulks and black is the difference between the two bulks (HT-LT).

Because regrowth in *Z. diploperennis* occurs through the re-activation of tillers, a trivial explanation for our results is that *reg1* and *reg3* simply promote strong tillering. To address this possibility, we performed a QTL-seq experiment utilizing high and low tiller number bulks from the field plot (Figure 5 and Supplementary Figure 2). The strongest enrichment of “Gigi” alleles in the high tiller bulk were observed on chromosomes 2 and 3, where multiple large-effect QTL have previously been mapped for tiller number (Westerbergh and Doebley, 2004; Chen et al., 2019, 2020). The region on chromosome 2 corresponding to *reg1* (44.6 Mb) showed a relatively low difference in SNP-index between high and low tiller bulks. There is also a tiller number QTL on chromosome 8, however, it is located at ∼164 Mb, a significant distance from *reg3* peak at 139.6 Mb. These data suggest that *reg1* and *reg3* are distinct from the major loci that control tiller number, and instead have specific roles in regrowth.

## Discussion

Previous genetic studies of perenniality in grasses have focused on morphological traits, most notably tiller and rhizome growth, and not the key phenotype of regrowth after senescence. Studies in rice (Hu et al., 2003; Fan et al., 2020), sorghum (Paterson et al., 1995; Kong et al., 2015), and maize (Westerbergh and Doebley, 2004) have highlighted that rhizome development is a complex, multigenic trait that is influenced by the environment. Rhizomes are organs necessary for over-wintering in grasses (Washburn et al., 2013) but since not all perennials must over-winter they may not be absolutely required for perennial regrowth.

In our experiments involving potted plants of *Z. diploperennis*, we did not detect rhizomes (data not shown), and neither did (Ma et al., 2019) who also worked primarily with potted plants. Rhizomes alone are unlikely to promote regrowth, and many perennial grasses regrow year after year without forming obvious rhizomes. Further, it is likely that the genetics of regrowth is simpler than the genetics of rhizome formation. A case in point is *Thinopyrum elongatum* which regrows from tillers alone (Lammer et al., 2004). When wheat lines containing individual *T. elongatum* chromosomes were screened for regrowth, just the addition of chromosome 4E was enough to confer perennial regrowth, implying that regrowth is under relatively simple, dominant genetic control (Lammer et al., 2004). Although a rudimentary form of perenniality can be achieved with regrowth alone, rhizomes are likely required for perennial plants to over-winter and sustain perennial regrowth in temperate environments.

The first two perennial regrowth QTL in *Z. diploperennis* were named *regrowth1* and *regrowth2* and lie near 33.0 Mb on chromosome 2 and 4.2 Mb on chromosome 7 using B73v4 coordinates (Ma et al., 2019). They were described as dominant, fully penetrant, and complementary to one another. However, the QTL corresponding to the *reg2* locus was not observed in either of our mapping populations (Figures 2–4 and Tables 3, 4). One explanation is that there was no significant polymorphism for this trait in our crosses involving P39 and Hp301. A QTL for tiller number called *tin1* was recently mapped to a location near *reg2* on chromosome 7. The high-tiller allele of this QTL is present in most sweet corn and popcorn lines but absent from other lines such as the inbred B73. The *tin1* polymorphism is a SNP that affects a splice-site to increase the transcript instability in a C2H2-zinc-finger transcription factor (Zhang et al., 2019). TIN1 controls tillering by directly repressing a previously identified tillering locus in maize, *grassy tillers1* (Whipple et al., 2011; Wills et al., 2013).

In contrast, our data strongly support the existence of a distinct and reproducible QTL peak corresponding to *reg1*, located on chromosome 2 at 43.8 Mb (in B73v4 coordinates). This peak is roughly 10 Mb away from the reported location of *reg1* in the B73 background (Ma et al., 2019). QTL positions are inherently approximations that are affected by population size, recombination rate, crossing partner, errors in genotyping and phenotyping, and effect size in a given environment. Advanced populations will be necessary for further fine-mapping to more accurately determine the position of *reg1*.

We also observed a QTL on chromosome 8 that we refer to as *regrowth3* (Figures 2, 3 and Table 2). Interestingly, *reg1* and *reg3* had their greatest impact at different stages of the growth cycle: *reg1* showed the highest LOD in the earlier months of January and February and this declined below the threshold of significance in May, while *reg3* showed its lowest LOD in January and reached its highest point in May. The fact that *reg3* exerted its strongest effects when regrowth was scored about 6 months after sowing could explain why it was not identified previously, since (Ma et al., 2019) scored regrowth soon after senescence. Similarly, we only scored early regrowth in our field study, and detected *reg1* but not *reg3*. A plausible scenario is that *reg1* promotes the initiation of regrowth while *reg3* is required for the continuation of regrowth.

We observed a lower percentage of regrown plants than what was reported in prior work (Ma et al., 2019). These authors generated two F_2_ populations using the maize inbred B73 that does not tiller and an heirloom landrace called Rhee Flint which develops numerous tillers. They reported 60% (B73-Zd) and 57% (Rhee Flint-Zd) regrowth following flowering and senescence, while we observed regrowth frequencies of 43 and 42% (P39/“Gigi” – greenhouse), 8% (Hp301/“Gigi” – greenhouse), and 17% (P39/“Gigi” – field) when scored at a similar developmental stage. Genotype-by-environment (G × E) interactions are well-known to play significant roles in determining plant phenotypes (Zeng et al., 1999; Yadav et al., 2003; Buescher et al., 2014; El-Soda et al., 2014; Frey et al., 2016; AlKhalifah et al., 2018; Li et al., 2018; McFarland et al., 2020). The same QTL are typically identified when grown in different environments, but they may vary in the magnitude of their effects (Buckler et al., 2009; Peng et al., 2011; Liu et al., 2014). Greenhouse and field study sites differ dramatically in a plant’s access to water, nutrients, microbes, growth space, and light and these factors likely contribute to the differences in regrowth rate we observed in these two environments. The maize parents used in the cross have a major impact on regrowth phenotype, as evidenced by the differences in regrowth observed in the P39 and Hp301 backgrounds (Table 3). Finally, *Z. diploperennis* is an outcrossing species and it is possible that the *Z. diploperennis* alleles in our crosses are not identical to those in the (Ma et al., 2019) study although the same accession was used.

Our results show *regrowth* QTL are distinct from those that regulate tiller number (Figures 2, 5) which mirrors the conclusion reached by Ma et al. (2019) that there was no significant association between tiller number and regrowth. Nevertheless, some degree of tillering is a prerequisite for perenniality (Galinat, 1981; Westerbergh and Doebley, 2004). A QTL corresponding to the known tiller number gene *tin1* on the short arm of chromosome 7 was not detected in our QTL-seq analysis, although it was found in studies where differences between teosinte and maize tiller numbers were considered (Chen et al., 2019, 2020). This QTL was also not detected in a population derived from a *Z. diploperennis* × *Z. mays* ssp. *parviglumis* cross (Westerbergh and Doebley, 2004), where both parents presumably have the high-tiller allele of *tin1*. Prior data show that the B73 inbred used by Ma et al. (2019) has the low-tiller *tin1* allele (Zhang et al., 2019) while the P39 inbred used in our study contains the high-tiller *tin1* allele. These data raise the possibility that the *reg2* locus (Ma et al., 2019), which we did not detect in our studies, is the tillering gene *tin1*, although further work will be required to test this possibility.

Branching architecture is regulated by numerous developmental and physiological cues including plant age and carbon status. Some of the known regulatory pathways may provide clues to how perennial grasses regulate tillering to achieve a perennial lifestyle. For instance, the microRNA miR156 is well-known to regulate juvenility in angiosperms (Wu et al., 2009) and to promote branching through a module affecting *SPL15*, *tb1*, and *gt1* (Chuck et al., 2007; Liu et al., 2017). Perennials rely on switches between juvenile and adult forms which are likely reflected in their miR156 levels. In *Arabis alpina*, a perennial closely related to Arabidopsis, miR156 levels remain high in some axillary meristem buds as plants age, which may affect the branching fate of these buds (Park et al., 2017). Sugar availability also positively affects tiller bud growth (Mason et al., 2014; Fichtner et al., 2017; Wang et al., 2020). In perennial grasses, mobilization of remaining sugars to the below-ground organs following has been well documented (Komor, 2000; Purdy et al., 2015). Trehalose 6-phosphate is derived from sucrose and serves as a signal of available sucrose levels (Figueroa and Lunn, 2016). Induction of axillary bud growth by sucrose is mediated by T6P (Fichtner et al., 2017; Dong et al., 2019) suggesting that the coordination of sucrose transport likely plays an important role in activating and/or maintaining regrowth following senescence.

What types of genes might underlie *regrowth* QTL? Proper spatiotemporal control of tiller regrowth for a perennial life strategy must utilize genes that respond to seasonal and physiological cues. Tillers form from basal axillary buds, and we understand much about how these buds are suppressed. A key regulator of tiller bud suppression is the TCP transcription factor encoded by *Teosinte branched-1* (*Tb1*) that dominantly initiates axillary bud dormancy (Doebley et al., 1995; Dong et al., 2019). *Tb1* coordinates cues from light quality (Kebrom et al., 2006), nutrient (Mason et al., 2014; Dong et al., 2019), age (Liu et al., 2017), and phytohormonal (Dong et al., 2019; Wang et al., 2019) pathways, suggesting many points where *Tb1* or downstream genes in this pathway may be modulated in the context of perennial regrowth. Although *Tb1* (on chromosome 1) was not detected as a QTL in our study, we speculate that *reg2* is caused by an allele of *tin1*. TIN1 promotes tiller bud growth by repressing *grassy tillers-1*, a downstream component of the *Tb1* pathway (Zhang et al., 2019). Future studies will focus on identifying *reg1* and *reg3*, and additional *regrowth* QTL to better understand the developmental processes of axillary bud dormancy and activation.

## Data Availability Statement

The datasets presented in this study can be found in online repositories. The names of the repository/repositories and accession number(s) can be found in the article/Supplementary Material.

## Author Contributions

RD oversaw the project. KS, DW, and RD wrote the manuscript. KS produced the figures, performed the QTL-seq and analysis, and QTL and statistical analyses. KS, HB, and DW grew, phenotyped, and collected tissue from plants. KS and HB extracted DNA and PCR genotyped. All authors contributed to the article and approved the submitted version.

## Conflict of Interest

The authors declare that the research was conducted in the absence of any commercial or financial relationships that could be construed as a potential conflict of interest.

## Publisher’s Note

All claims expressed in this article are solely those of the authors and do not necessarily represent those of their affiliated organizations, or those of the publisher, the editors and the reviewers. Any product that may be evaluated in this article, or claim that may be made by its manufacturer, is not guaranteed or endorsed by the publisher.

## Footnotes

1
http://broadinstitute.github.io/picard/

## Supplementary Material

Supplementary Figure 1 Distribution of filtered homozygous “Gigi” SNPs mapped to P39v1 reference assembly.

Supplementary Figure 2 Distribution of tiller number phenotype in field-grown P39/“Gigi” F_2_ plants. Red and blue shaded regions indicate plants that were included in low and high tiller QTL-seq bulks, respectively.

Supplementary Table 1 List of Illumina sequenced samples for this study. SE150, Single End 150 sequencing; PE150, Paired End 150 sequencing.

Supplementary Table 2 Number of filtered, homozygous SNPs per chromosome present in *Z. diploperennis* “Gigi” from Illumina reads aligned to maize P39 reference.

Supplementary File 1 Marker and PCR primer information. This file contains primer names, sequences, and positions in references P39, Hp301, and B73v4.

Supplementary File 2 Genotypes for P39/“Gigi” F_2_ regrown plants in.csv format that can be read into R/qtl used to generate Figure 3. Regrowth data were also used to generate Table 3. A, “Gigi” homozygote; H, heterozygote; B, P39 homozygote. The first four columns display regrowth phenotype information, where 1, regrowth; 0, non-regrowth.

Supplementary File 3 Genotypes for Hp301/“Gigi” F_2_ regrown plants in.csv format that can be read into R/qtl used to generate Figure 4. Regrowth data were also used to generate Table 3. A, “Gigi” homozygote; H, heterozygote; B, Hp301 homozygote. The first four columns display regrowth phenotype information, where 1, regrowth; 0, non-regrowth.

Supplementary File 4 Regrowth phenotypes of P39/“Gigi” F_2_ plants used to generate Table 1.

Supplementary File 5 Genotypes for P39/“Gigi” F_2_ regrown plants used to generate Table 2. Z, “Gigi” homozygote; H, heterozygote; P, P39 homozygote.

Supplementary File 6 Genotypes and regrowth phenotypes for P39/“Gigi” F_2_ regrown plants used to generate Table 4. Z, “Gigi” homozygote; H, heterozygote; P, P39 homozygote. For the “regrowth” phenotype, 1, regrowth; 0, non-regrowth.
