# Supplementary material for: QTL Map of Early- and Late-Stage Perennial Regrowth in Zea diploperennis
Source: Front Plant Sci. 2021 Aug 24;12:707839. doi: 10.3389/fpls.2021.707839 (PMC8421791; doi:10.3389/fpls.2021.707839)
Supplement: Supplementary Figure 1 — Distribution of filtered homozygous “Gigi” SNPs mapped to P39v1 reference assembly. [file Data_Sheet_1.zip › Supplementary Table 1.DOCX]

**Supplementary Table 1: List of Illumina sequenced samples for this study.** SE150=Single End 150 sequencing; PE150=Paired End 150 sequencing.

| **Sample** | **Sequencing Type** | **Reads/Read Pairs** | **Sequencer** | **SRA Accession** |
| --- | --- | --- | --- | --- |
| *Z. diploperennis* ‘Gigi’ | PE150 | 449,924,498 | NextSeq 500 | SAMN17833852 |
| Regrowth Bulk | SE150 | 246,314,039 | HiSeq 4000 | SAMN17833853 |
| Non-regrowth Bulk | PE150 | 21,841,531 | NextSeq 500 | SAMN17833854 |
| Non-regrowth Bulk | PE150 | 374,940,596 | HiSeq 4000 | SAMN17833854 |
| High-tiller Bulk | PE150 | 325,222,732 | HiSeq 4000 | SAMN17833855 |
| Low-tiller Bulk | PE150 | 330,008,372 | HiSeq 4000 | SAMN17833856 |
