# Supplementary material for: QTL Map of Early- and Late-Stage Perennial Regrowth in Zea diploperennis
Source: Front Plant Sci. 2021 Aug 24;12:707839. doi: 10.3389/fpls.2021.707839 (PMC8421791; doi:10.3389/fpls.2021.707839)
Supplement: Supplementary Figure 1 — Distribution of filtered homozygous “Gigi” SNPs mapped to P39v1 reference assembly. [file Data_Sheet_1.zip › Supplementary Table 2.docx]

**Supplementary Table 2: Number of filtered, homozygous SNPs per chromosome present in *Z. diploperennis* 'Gigi' from Illumina reads aligned to maize P39 reference.**

| **Chromosome** | **Number of SNPs** |
| --- | --- |
| chr1 | 294,198 |
| chr2 | 256,571 |
| chr3 | 267,237 |
| chr4 | 338,163 |
| chr5 | 222,403 |
| chr6 | 172,588 |
| chr7 | 124,049 |
| chr8 | 165,662 |
| chr9 | 205,810 |
| chr10 | 133,573 |
| Total | 2,180,254 |
