# Supplementary figures and images for: QTL Map of Early- and Late-Stage Perennial Regrowth in Zea diploperennis
Source: Front Plant Sci. 2021 Aug 24;12:707839. doi: 10.3389/fpls.2021.707839 (PMC8421791; doi:10.3389/fpls.2021.707839)

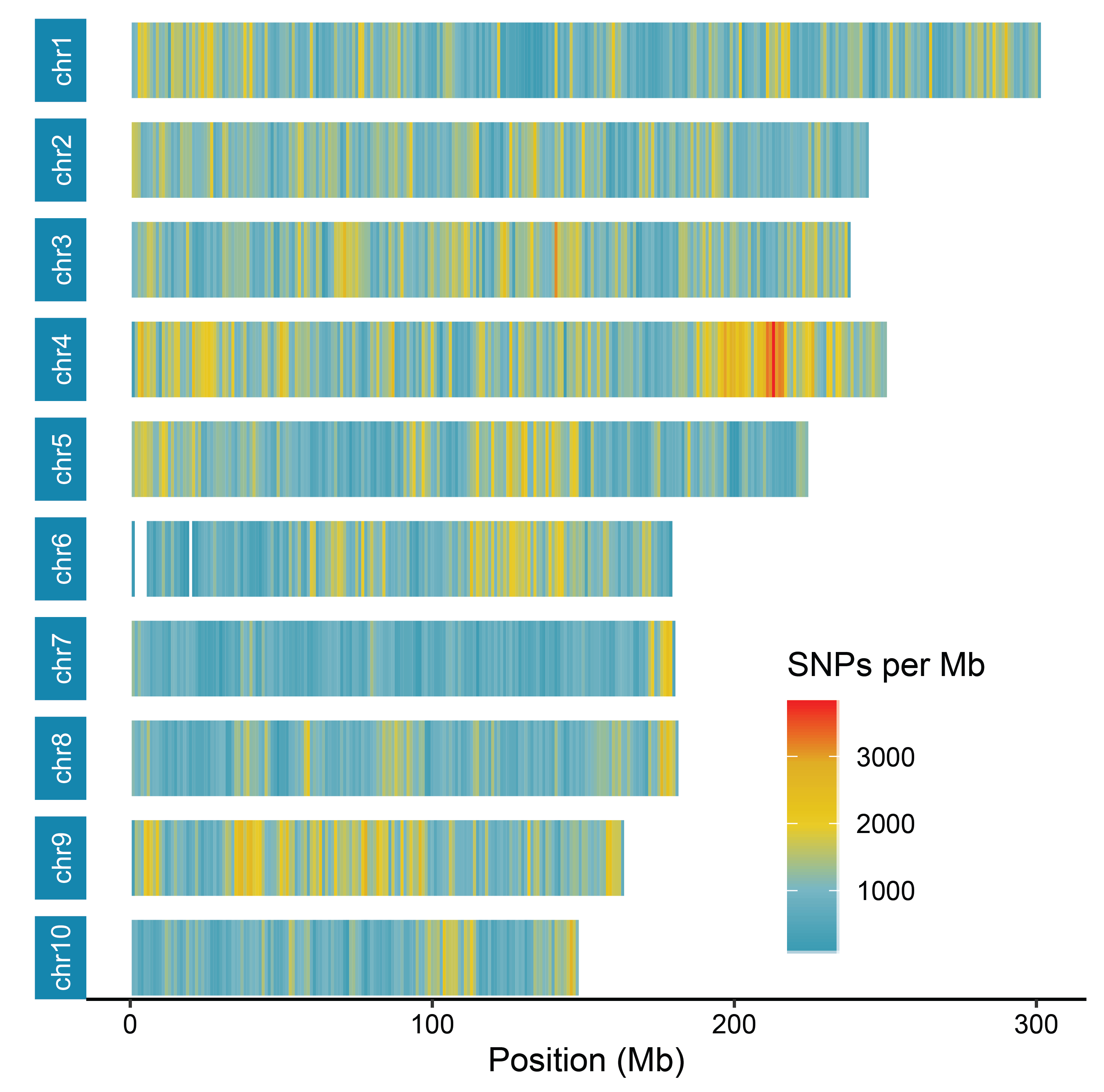

Supplement: Supplementary Figure 1 — Distribution of filtered homozygous “Gigi” SNPs mapped to P39v1 reference assembly. [file Data_Sheet_1.zip › Supplementary Figure 1.TIF]

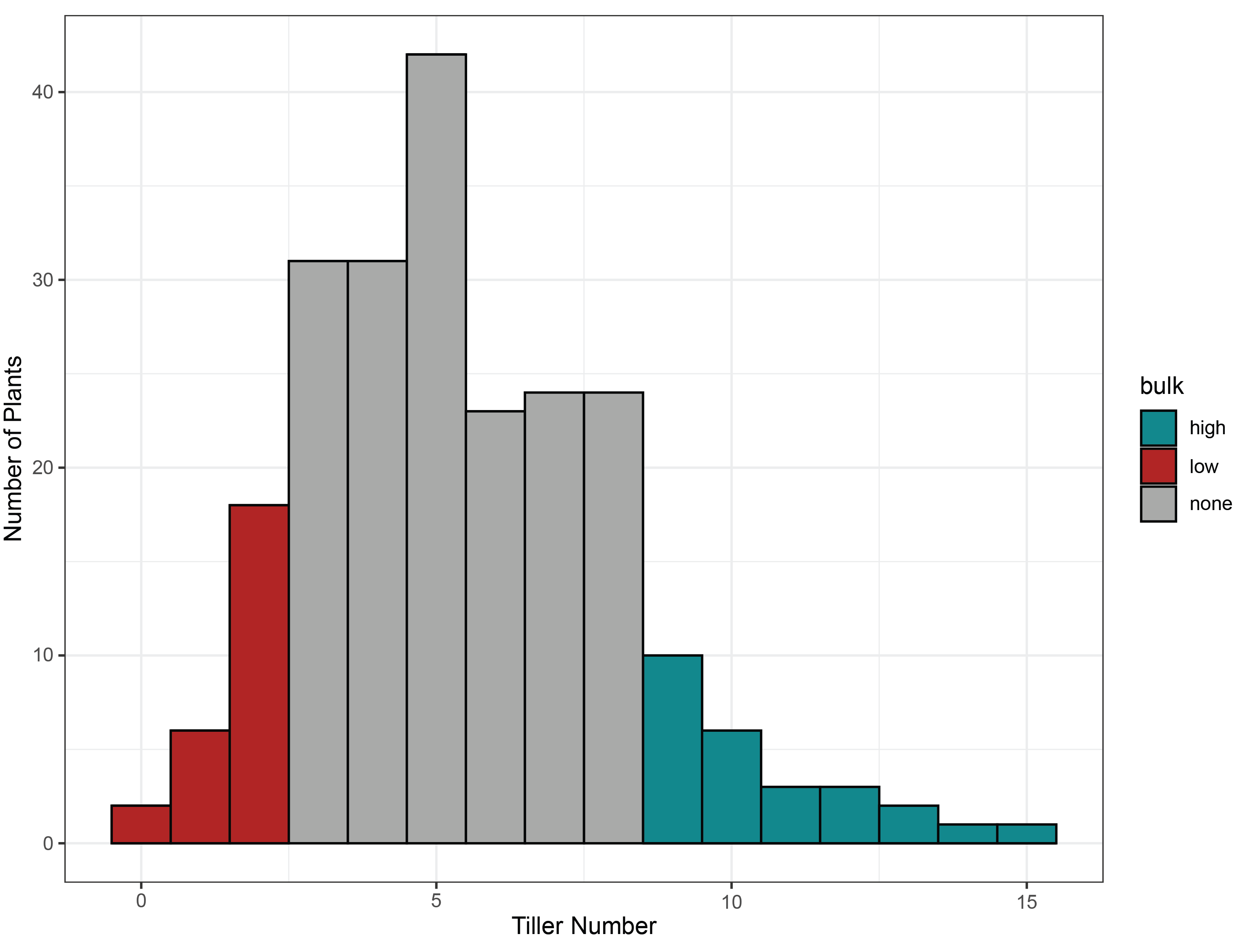

Supplement: Supplementary Figure 1 — Distribution of filtered homozygous “Gigi” SNPs mapped to P39v1 reference assembly. [file Data_Sheet_1.zip › Supplementary Figure 2.TIF]
